# Bacterial Etiology of Bloodstream Infections and Antimicrobial Resistance Patterns from a Tertiary Care Hospital in Malé, Maldives

**DOI:** 10.1155/2021/3088202

**Published:** 2021-10-18

**Authors:** Aishath Maharath, Mariyam Shabeena Ahmed

**Affiliations:** ^1^Faculty of Health Sciences, The Maldives National University, Malé 20371, Maldives; ^2^Research Center, The Maldives National University, Malé 20371, Maldives

## Abstract

**Background:**

Bloodstream infections pose a significant health problem worldwide and is a major cause of morbidity and mortality in many countries. It is important to have country-specific data for major pathogens causing bloodstream infections, in light of emerging resistance patterns of common bacterial isolates. Due to the scarcity of reports in this area, the aim of this study was to identify bacterial pathogens causing bloodstream infections among the study population.

**Methods:**

A retrospective analysis of blood culture samples received at the Department of Laboratory Medicine, Indira Gandhi Memorial Hospital, Malé, Maldives, was performed for reports between January 2016 and December 2017.

**Results:**

Out of the 471 culture-positive samples, 278 (59%) were males and 193 (41%) were females. Amongst the culture-positive samples, 338 (71.8%) Gram-positive organisms were isolated and 133 (28.2%) Gram-negative organisms were isolated. Coagulase-negative *Staphylococcus* (CoNS) was the most frequently isolated blood-borne bacterial pathogen in this study, accounting for 53.6% and 50.9% of the isolates in 2016 and 2017, respectively. Other frequently isolated pathogens included *Staphylococcus aureus* (15.9% and 10.3%), *Klebsiella* spp. (10.5% and 16.4%), and *Escherichia coli* (7.1% and 10.8%). Coagulase-negative *Staphylococcus* (CoNS) revealed high percentage of resistance among the tested antimicrobials, ampicillin, cephalexin, cefotaxime, and gentamicin. Over the two years, a significant difference between the percentage resistance among paediatric and adult patients was observed for coagulase-negative *Staphylococcus* (CoNS) isolate resistance to ampicillin (*p* ≤ 0.001), cephalexin (*p* ≤ 0.001), cefotaxime (*p* ≤ 0.001), gentamicin (*p*=0.008), and cotrimoxazole (SXT) (*p* ≤ 0.001). When comparing the significant antimicrobial resistance trends, it can be seen that Enterobacteriaceae isolates also demonstrated high resistance to ampicillin and gentamicin as well as second- and third-generation cephalosporins.

**Conclusions:**

This study highlights the major bacterial pathogens involved in bloodstream infections in the healthcare setting of Malé, Maldives, and antibiotic resistance patterns. The results indicate that further characterization of bacteremia and its resistance patterns is needed to combat bloodstream infections.

## 1. Introduction

Bloodstream infections (BSI) are defined broadly as the presence of viable microorganisms in the blood, which can lead to inflammation in the host and alter the clinical and hemodynamic properties and lead to morbid consequences [1]. The presence of microorganisms, however, transiently in the circulation poses a threat to most organs. The consequences of bloodstream infections if not treated can lead to shock, disseminated intravascular coagulation (DIC), multiple organ failure, and death [2]. Bloodstream infections are a major public health problem worldwide, and it has been associated with significant morbidity and mortality [3]. Although it is still common in developed nations, the burden is high in the least developed and developing countries [4].

There is a considerable variation in epidemiology and pathogen profile of microorganisms, which cause BSI [5]. Population-based studies originating in countries such as Australia, Canada, Denmark, Finland, Iceland, New Zealand, Sweden, and USA show the etiologies of BSI to be mainly from the organisms *Escherichia coli*, *Staphylococcus aureus*, and *Streptococcus pneumoniae* [3]. In contrast to this, the pathogen profiles vary in Africa and Asia. *Salmonella enterica* has been implicated as one of the major pathogens causing BSI in both African and Asian nations [6].

The potential outcomes of BSI and the delays in performing and receiving culture results often lead to empirical treatment. In developing countries, this may also be due to the lack of treatment guidelines and unavailability of susceptibility patterns for local isolates [3]. Even in constrained healthcare settings, the knowledge of local antibiotic resistance patterns will help in selecting effective empirical therapy [7]. As such there is an emerging trend of BSI caused by Gram-negative organisms and an increased incidence of drug-resistant strains [8].

In light of the growing trends of BSI infections globally and emerging antimicrobial resistance profiles of implicated organisms, it is important to conduct studies to investigate the pathogen profiles for Maldives. In addition to this, published data for the country is limited and there is a need for baseline studies in this area. Therefore, a retrospective study was conducted to determine the common bacterial agents associated with bloodstream infections and their antibiotic resistance patterns.

## 2. Methods

In this retrospective study, blood culture sample results were obtained for both out- and inpatients who attended the Indira Gandhi Memorial Hospital, which is a tertiary hospital located in Malé, the capital city of the Republic of Maldives. This is a 350-bed government hospital which is located in the population dense capital island. A total of 9556 blood sample results from January 2016 to December 2017 were processed. Oxoid SIGNAL (Thermo Fisher Scientific Inc.) blood culture systems were used to identify the presence of pathogens.

Collected blood culture samples were inoculated into Oxoid SIGNAL (Thermo Fisher Scientific Inc.) blood culture system and incubated at 37°C for 7 days or until the presence of a pathogen is indicated from the Oxoid SIGNAL blood culture system. Positive culture samples were directly inoculated into MacConkey agar and blood agar plates. The plates were incubated aerobically at 37°C and examined after 18–24 hrs. Bacterial organisms were isolated using standard bacteriological procedures based on the current Clinical and Laboratory Standards Institute (CLSI) 2014–2017 guideline.

Antimicrobial susceptibility tests were carried out by using the disk diffusion method (modified Kirby–Bauer method) on Mueller–Hinton agar (HiMedia Laboratories, India). Antibiotics that were used in this study include ampicillin (10 *μ*g), ampicillin-sulbactam (10/10 *μ*g), amoxicillin-clavulanate (20/10 *μ*g), piperacillin-tazobactam (100/10 *μ*g), cephalexin (30 *μ*g), cefuroxime (30 *μ*g), cefotaxime (30 *μ*g), ceftazidime (30 *μ*g), ceftriaxone (30 *μ*g), cefepime (30 *μ*g), imipenem (10 *μ*g), meropenem (10 *μ*g), gentamicin (10 *μ*g), tobramycin (10 *μ*g), amikacin (30 *μ*g), netilmicin (30 *μ*g), ciprofloxacin (5 *μ*g), cotrimoxazole (1.25/23.75 *μ*g), and colistin (30 *μ*g) from HiMedia Laboratories, India. The ATCC cultures *Staphylococcus aureus* ATCC 25923 and *Escherichia coli* ATCC 25218 were used as control strains.

In this study, multidrug resistance (MDR) was categorized as acquired nonsusceptibility to at least three classes of antibiotics [9].

### 2.1. Ethical Considerations

Ethical clearance was obtained from the Maldives National University as well as National Health Research Committee, Ministry of Health, Malé, Maldives. Patient data was kept anonymous throughout the study and kept confidential.

### 2.2. Statistical Analysis

Culture positivity was seen in 471(4.9%) samples, and 9085(95.1%) samples were negative. The 471 culture positive samples were entered into the Statistical Package for Social Sciences (SPSS) version 20.0, and all the analysis was carried out using this program.

The data were analyzed using chi-square (*χ*^2^), independent samples *t*-test, and frequency distributions. *χ*^2^ was conducted to find out the significant difference between the bacterial isolates related to age groups over the two years. *t*-test was carried out to find out the significant difference between the percentage resistance related to individual microbials for each bacterial isolate. These analyses were carried out with 95% confidence interval (CI) and a *p* value less than 0.05 was regarded as significant. The frequency distribution was used to compare the difference in BSI among male, female, and age groups over the years.

## 3. Results

Out of the 471 culture-positive samples, 278 (59%) were males and 193 (41%) were females. Amongst the culture-positive samples, 338 (71.8%) Gram-positive organism were isolated and 133 (28.2%) Gram-negative organisms were isolated. The data contained specimens obtained from paediatric patients (*n* = 130, 27.6%) and adult patients (*n* = 341, 72.4%) (Table 1).

Table 2 shows the percentage distribution of organism isolated from blood culture samples during the two-year study period. Coagulase-negative *Staphylococcus* (CoNS) was the most frequently isolated blood-borne bacterial pathogen in this study. Other frequently isolated pathogens included *Staphylococcus aureus*, *Klebsiella* spp., and *Escherichia coli*.

As illustrated in Table 3, Gram-positive bacteria (71.8%) were the most common pathogenic agents compared to the Gram-negative bacteria (28.2%) in this study (*χ*^2^=5.533; *p*=0.019).  Table 4 details the common bacterial isolates causing BSI among the two group of patients. Predominant isolates among Gram-positive isolates were coagulase-negative *Staphylococcus (CoNS)* 246 (52.2%), *Staphylococcus aureus* 62 (13.2%), *Streptococcus* spp. 23 (4.9%), and *Enterococcus* spp. 7 (1.5%). Among the Gram-negative isolates, the most predominant was *Klebsiella* spp. 63 (13.4%) followed by *Escherichia coli* 42 (8.9%), *Pseudomonas* spp. 12 (2.5%), *Acinetobacter* spp. 7(1.5%), *Pseudomonas aeruginosa* 5 (1.1%), and *Salmonella* spp. 4 (0.8%).

Based on the age categories, the data contained specimens obtained from neonates (*n* = 71), infants (*n* = 21), children (*n* = 38), adults (*n* = 163), and elderly (*n* = 176) (Table 4). During the two years of data collection, more adult patients were found with BSI (72.4%) as compared to paediatric patients (27.6%). But this trend was not statistically significant (*p*=0.063) (Table 5).

When compared between the two years, a significant difference is seen in adults (31.8%, 38.4%; *p*=0.017). Among the paediatric group, neonates comprised a high proportion of culture positive samples (15.1%) in comparison to infants (4.5%) and children (8.1%). Elderly comprised dominant culture positive samples (37.4%) in comparison to adults (34.6%). Amongst all cases, the highest proportion of positive BSI was seen among the elderly (37.4%) (Table 6).

When considering the trends of antimicrobial susceptibilities of Gram-positive isolates in the study, coagulase-negative *Staphylococcus* (CoNS) revealed high level of resistance among tested antimicrobials over the two years (Table 7). Over the two years, a significant difference between the percentage resistance among paediatric and adult patients was observed for coagulase-negative *Staphylococcus* (CoNS) isolate resistance to ampicillin (*p* ≤ 0.001), cephalexin (*p* ≤ 0.001), cefotaxime (*p* ≤ 0.001), gentamicin (*p*=0.008), and cotrimoxazole (SXT) (*p* ≤ 0.001). *Staphylococcus aureus* has also shown significant difference in the percentage resistance among the two group of patients to ampicillin (*p* ≤ 0.001), cephalexin (*p* ≤ 0.001), and ciprofloxacin (*p*=0.005).

Both Tables 8 and 9 show the trend of antimicrobial resistance in Gram-positive and Gram-negative isolates over the two years of study period, respectively. Among Gram-negative isolates in the study, *Klebsiella* spp. revealed high level of resistance among tested antimicrobials over the two years (Table 9). Over the two years, a significant difference was observed for *Klebsiella* spp. resistance to ampicillin (*p* ≤ 0.001), ampicillin/sulbactam (*p* ≤ 0.001), piperacillin/tazobactam (*p* ≤ 0.001), cephalexin (*p*=0.025), cefuroxime (*p*=0.004), cefotaxime (*p*=0.032), ceftriaxone (*p*=0.010), cefepime (*p* ≤ 0.001), and ciprofloxacin (*p*=0.034) (Table 9). *Escherichia coli* isolates showed significant resistance to antimicrobials ampicillin (*p*=0.001), cephalexin (*p*=0.022), cefuroxime (*p*=0.018), cefotaxime (*p*=0.005), ceftazidime (*p*=0.041), ceftriaxone (*p*=0.046), and ciprofloxacin (*p*=0.038). Similarly, *Acinetobacter* spp. showed significant resistance to cephalosporins cefotaxime (*p*=0.010), ceftriaxone (*p*=0.010), and aminoglycoside gentamicin (*p*=0.010) (as well as fluoroquinolone and ciprofloxacin (*p*=0.010)).

Trends of multidrug-resistant (MDR) isolates showed that coagulase-negative *Staphylococcus* (CoNS) was comparatively higher at 45.2% (Table 10). *Klebsiella* spp. at 31.3% MDR isolates had a statistically significant increase when compared between the two years of study.

## 4. Discussion

The data showed a variation in bloodstream infections (BSI) between age groups. The elderly comprised the dominant culture-positive group (37.4%) in comparison to the adult population. Neonates comprised a high proportion of culture positive cases (15.1%) amongst the paediatric group. It is interesting to see culture-positive samples in extremes of ages, and the reason for this distribution is not clear as risk factors for development of blood culture infection have not been evaluated in this particular study.

Studies have implicated a higher risk for neonates for central line-associated bloodstream infections, which comprise a significant component of healthcare-associated infections [10]. This may be due to host risk factors mainly relative to immunodeficiency and breach of physical barriers or due to contamination during access of the catheters [11]. The major organisms implicated in central line-associated bloodstream infections are routine skin colonizers, namely, coagulase-negative *Staphylococcus* (CoNS), *Staphylococcus aureus*, and *Candida* species [12].

In this study,71.8% of bloodstream infections were caused by Gram-positive bacteria and 28.2% by Gram-negative bacteria. Similar findings were seen from studies by Shrestha et al., Nepal [13], Arora et al., India [14], Moyo et al., Tanzania [15], and Takeshita et al., Vietnam [16]. In contrast to this, Gram-negative bacteria have been implicated as the commonest cause of BSI in studies by Parajuli et al., Nepal [17], Khurana et al., India [18], Dramowski et al., South Africa [19], and Easow et al., Nepal [20].

Among the Gram-positive organisms, coagulase-negative *Staphylococcus* (CoNS) (52.2%) was the most common bacterial pathogen causing BSI in this study. Although there was no statistically significant variation between the paediatric and adult populations, paediatric isolates should be dealt with care and there is a need to differentiate between contaminants and true pathogens. Although coagulase-negative *Staphylococcus* (CoNS) was noted as contaminants in the past [21], studies have shown that coagulase-negative *Staphylococcus* (CoNS) and viridans group *Streptococcus* are frequently associated with immunocompromised paediatric bloodstream infections [22, 23]. Numerous studies have been carried out on the process of ruling out contaminants from pathogens among coagulase-negative *Staphylococus* [24, 25]. Studies comparing low- and middle-income countries amongst Asia and Africa regions have showed variation in the causative organisms implicated in BSI in paediatric populations [26, 27]. Meta-analysis by Droz et al. [28] showed that Gram-negative bacteria accounted for 63.9% of BSI and *Salmonella* spp. was the most common pathogen in Asia, while *S*. *aureus* and *S*. *pneumoniae* were most predominant in Africa. Gram-positive bacteria were more likely seen as causative organism in paediatric BSI in high-income countries [29]. This variation could be due to epidemiological differences of causative organisms. *Staphylococcus aureus* (13.2%) was the second most common isolated organism in the Gram-positive category followed by *Streptococcus* spp. (4.9%).

*Klebsiella* spp. (13.4%) was the predominant organism isolated among Gram-negative bacteria, followed by *Escherichia coli* (8.9%), *Pseudomonas* spp. (12.5%), *Acinetobacter* spp. (1.5%), *Pseudomonas aeruginosa* (1.1%), and *Salmonella* spp. (0.8%). It is interesting to note that *Salmonella* spp. constitute a prominent pathogen in BSI in studies carried out in the South East Asia region, Bangladesh [30, 31], Nepal [32, 33], India [34], and Pakistan [35], but in this study it is not significant. This decrease comparatively could be due to improved urban water management and public health in Maldives. This variation may be due to difference in geographical location and endemic variation. When comparing the significant antimicrobial resistance trends, it can be seen that Enterobacteriaceae isolates demonstrated high resistance to ampicillin and gentamicin as well as second- and third-generation cephalosporins.

Cephalosporins have been used in many settings as empirical therapy [36] mainly due to their low toxicity, broad spectrum activity, and high effectiveness. In this setting, the previous antimicrobial usage and clinical data for the cases have not been evaluated alongside resistance trends. However, the antimicrobial resistance patterns seen here are significant in light of Class C cephalosporinases (Amp C), cabapenemases (including metallo-beta lactamases), and extended spectrum *β*-lactamases (ESBL) being the main mechanism for cephalosporin resistance [37]. Further studies with phenotyping are required to generate antibiogram for Gram-positive and Gram-negative isolates. In addition to this, evaluating the extent of resistant isolates can help mitigate the impact of antimicrobial resistance in healthcare settings.

The main limitation of this study, as it was retrospective in nature, was the inability to determine standardization of techniques for individual cases. The other limitation is that comprehensive clinical data and recent antibiotic usage has not been documented. Also, as this study was performed in a single center, it cannot be generalized for the whole population of Maldives.

## 5. Conclusions

In conclusion, this study provides a baseline insight into the bacterial aetiology of bloodstream infections both in paediatric and adult populations. Gram-positive organisms are the major contributors to bloodstream infections in this study. Nevertheless, Gram-negative organisms demonstrated a high percentage of antimicrobial resistance, which needs to be further elucidated. In addition to this, we hope this study would help researchers and policymakers to prioritize respective research options in light of global challenges in combatting antimicrobial resistance.

## Figures and Tables

**Table 1:** Trends in the organisms isolated from the culture-positive patient samples.

Organisms	Bacterial isolate (%)	Paediatric (*n* = 130)		Adult (*n* = 341)	
		Male (*n* = 80)	Female (*n* = 50)	Male (*n* = 198)	Female (*n* = 143)
Gram-positive	338 (71.8)	68 (85.0)	39 (78.0)	136 (68.7)	95 (66.4)
Gram-negative	133 (28.2)	12 (15.0)	11 (22)	62 (31.3)	48 (33.6)

**Table 2:** Distribution of organism isolated from blood culture samples during the two years.

Bacterial isolates	Number (%)	Year	
		2016	2017
*Streptococcus* spp.	23 (4.9)	14 (5.9)	9 (3.9)
Coagulase-negative *Staphylococcus* (CoNS)	246 (52.2)	128 (53.6)	118 (50.9)
*Staphylococcus aureus*	62 (13.2)	38 (15.9)	24 (10.3)
*Enterococcus* spp.	7 (1.5)	3 (1.3)	4 (1.7)
*Klebsiella* spp.	63 (13.4)	25 (10.5)	38 (16.4)
*Acinetobacter* spp.	7 (1.5)	4 (1.7)	3 (1.3)
*Pseudomonas aeruginosa*	5 (1.1)	3 (1.3)	2 (0.9)
*Pseudomonas* spp.	12 (2.5)	4 (1.7)	8 (3.4)
*Escherichia coli*	42 (8.9)	17 (7.1)	25 (10.8)
*Salmonella* spp.	4 (0.8)	3 (1.3)	1 (0.4)
Total	471	239	232

**Table 3:** Trends of Gram-positive and Gram-negative bacterial isolates.

	Number (%)	2016 (*n* = 239)	2017 (*n* = 232)	*χ* ^2^	*p* value
Gram-positive	338 (71.8)	183 (76.6)	155 (66.8)	5.533	0.019
Gram-negative	133 (28.2)	56 (23.4)	77 (33.2)		

**Table 4:** Trends of bacterial isolates among paediatric and adult populations, *n*=(471).

Bacterial isolates	Number (%)	Paediatric patients (*n* = 130)		Adult patients (*n* = 341)		*p* value
		2016 (*n*=75)	2017 (*n*=55)	2016 (*n*=164)	2017 (*n*=177)	
Gram-positive isolates	338 (71.8%)	61 (81.3)	46 (83.6)	122 (74.4)	109 (61.6)	
*Streptococcus* spp.	23 (4.9)	8 (10.7)	5 (9.1)	6 (3.7)	4 (2.3)	0.940
Coagulase-negative *Staphylococcus* (CoNS)	246 (52.2)	49 (65.3)	35 (63.6)	79 (48.2)	83 (46.9)	0.154
*Staphylococcus aureus*	62 (13.2)	3 (4.0)	6 (10.9)	35 (21.3)	18 (10.2)	0.063
*Enterococcus* spp.	7 (1.5)	1 (1.3)	0 (0.0)	2 (1.2)	4 (2.3)	0.212
Gram-negative isolates	133 (28.2)	14 (18.7)	9 (16.4)	42 (25.6)	68 (38.4)	
*Klebsiella* spp.	63 (13.4)	6 (8.0)	6 (10.9)	19 (11.6)	32 (18.1)	0.417
*Acinetobacter* spp.	7 (1.5)	4 (5.3)	3 (5.5)	4 (2.4)	3 (1.7)	—
*Pseudomonas aeruginosa*	5 (1.1)	1 (1.3)	0 (0.0)	2 (1.2)	2 (1.1)	0.361
*Pseudomonas* spp.	12 (2.5)	3 (4.0)	1 (1.8)	1 (0.6)	7 (4.0)	0.030
*Escherichia coli*	42 (8.9)	3 (4.0)	1 (1.8)	14 (8.5)	24 (13.6)	0.139
*Salmonella* spp.	4 (0.8)	1 (1.3)	1 (1.8)	2 (1.2)	0 (0.0)	0.248

**Table 5:** Trends of group of patients associated with BSI.

Patient group	Number (%)	2016 (*n* = 239)	2017 (*n* = 232)	*χ* ^2^	*p* value
Paediatric	130(27.6)	75(31.4)	55(23.7)	3.469	0.063
Adult	341(72.4)	164(68.7)	177(76.2)		

**Table 6:** Trends of BSI among various group of patients

Patient group	Age category	Age group	Number (%)	2016 (*n* = 239)	2017 (*n* = 232)	*p* value
Paediatrics	Neonates	<28 days	71 (15.1)	44 (18.4)	27 (11.6)	0.549
	Infants	>28 days to 1 year	21 (4.5)	11 (4.6)	10 (4.3)	0.223
	Children	1–15 years	38 (8.1)	20 (8.4)	18 (7.8)	0.239

Adults	Adults	16–65 years	163 (34.6)	76 (31.8)	89 (38.4)	**0.017**
	Elderly	>65 years	176 (37.4)	88 (36.9)	88 (37.9)	0.589

Note: *p* values in bold indicate significant effects.

**Table 7:** Antimicrobial resistance of Gram-positive isolates among paediatric and adult patients over the two years.

Antimicrobial agents	*Streptococcus* spp.				*p* value	Coagulase-negative *Staphylococcus* (CoNS)				*p* value	*Staphylococcus aureus*				*p* value	*Enterococcus* spp.				*p* value
	Resistance (%)					Resistance (%)					Resistance (%)					Resistance (%)				
	2016		2017			2016		2017			2016		2017			2016		2017		
	Paediatric	Adult	Paediatric	Adult		Paediatric	Adult	Paediatric	Adult		Paediatric	Adult	Paediatric	Adult		Paediatric	Adult	Paediatric	Adult	
Ampicillin	0 (0)	0 (0)	0 (0)	0 (0)	—	36 (73)	57 (72)	21 (62)	64 (77)	**≤0.001**	3 (100)	29 (85)	6 (100)	13 (76)	**≤0.001**	0 (0)	0 (0)	—	0(0)	—
Piperacillin/tazobactam	0 (0)	0 (0)	0 (0)	0 (0)	—	0 (0)	1 (7)	0 (0)	—	—	0 (0)	0 (0)	—	—	—	0 (0)	0 (0)	—	0(0)	—
Cloxacillin	0 (0)	0 (0)	—	0 (0)	—	6 (16)	8 (15)	1 (7)	3 (11)	0.535	0 (0)	0 (0)	0 (0)	0 (0)	—	—	—	—	-	—
Cephalexin	0 (0)	1 (20)	1 (20)	0 (0)	—	9 (19)	30 (40)	8 (25)	28 (37)	**≤0.001**	1 (33)	12 (39)	1 (17)	7 (47)	**≤0.001**	1 (100)	1 (50)	—	2(67)	0.073
Cefuroxime	0 (0)	0 (0)	—	—	—	1 (50)	0 (0)	0 (0)	—	—	—	—	—	1 (50)	—	—	—	—	—	—
Cefotaxime	0 (0)	0 (0)	0 (0)	0 (0)	—	9 (20)	25 (42)	8 (25)	24 (32)	**≤0.001**	1 (50)	9 (29)	1 (17)	4 (29)	0.363	1 (100)	0 (0)	—	—	—
Ceftazidime	—	—	—	—	—	—	—	2 (100)	—	—	—	—	—	—	—	—	—	—	—	—
Ceftriaxone	0 (0)	0 (0)	0 (0)	0 (0)	—	1 (50)	3 (42)	0 (0)	1 (100)	0.835	—	1 (100)	—	1 (100)	-	-	0 (0)	-	-	-
Cefepime	—	—	—	—	—	—	—	—	—	—	—	—	—	—	—	—	—	—	—	—
Vancomycin	—	—	—	—	—	0 (0)	0 (0)	0 (0)	0 (0)	—	—	0 (0)	—	0(0)	—	—	—	—	—	—
Gentamicin	4 (57)	3 (75)	1 (50)	0 (0)	**≤0.001**	9 (21)	23 (36)	8 (35)	12 (26)	**0.008**	0 (0)	4 (15)	0 (0)	1(11)	-	1(100)	1(50)	-	2(67)	0.073
Tobramycin	—	—	—	—	—	—	—	—	—	—	—	—	—	—	—	—	—	—	—	—
Amikacin	—	1 (100)	—	—	—	0 (0)	0 (0)	0 (0)	0 (0)	—	0 (0)	0 (0)	0 (0)	0(0)	—	—	—	—	1(100)	—
Netilmicin	—	—	—	—	—	—	0 (0)	—	—	—	—	—	—	—	—	—	—	—	—	—
Ciprofloxacin	0 (0)	1 (25)	2 (40)	1 (25)	—	9 (21)	25 (38)	7 (23)	20 (27)	—	1 (33)	10 (37)	0 (0)	6(40)	**0.005**	1(100)	1(50)	—	2(67)	0.073
Cotrimoxazole	3 (50)	2 (40)	1 (50)	3 (75)	0.295	10 (22)	22 (30)	4 (13)	12 (18)	**≤0.001**	0 (0)	3 (10)	0 (0)	1(7)	—	1(100)	1(50)	—	1(100)	0.667

Note: *p* values in bold indicate significant effects.

**Table 8:** Antimicrobial resistance pattern of Gram-positive isolates among two-year study period.

Antimicrobial agents	*Streptococcus* spp. (*n* = 23)			Coagulase-negative *Staphylococcus* CoNS (*n* = 246)			*Staphylococcus aureus* (*n* = 62)			*Enterococcus* spp. (*n* = 7)		
	Resistance (%)		*p* value	Resistance (%)		*p* value	Resistance (%)		*p* value	Resistance (%)		*p* value
	2016 (*n* = 14)	2017 (*n* = 9)		2016 (*n* = 128)	2017 (*n* = 118)		2016 (*n* = 38)	2017 (*n* = 24)		2016 (*n* = 3)	2017 (*n* = 4)	
Ampicillin	0 (0)	0 (0)	—	93 (73)	85 (73)	0.999	32 (86)	19 (83)	**0.038**	0 (0)	0 (0)	—
Piperacillin/tazobactam	0 (0)	0 (0)	—	1 (5)	0 (0)	0.826	0 (0)	—	—	0 (0)	0 (0)	—
Cloxacillin	0 (0)	0 (0)	—	14 (16)	4 (10)	0.695	0 (0)	0 (0)	—	—	—	—
Cephalexin	1 (8)	1 (11)	0.675	39 (32)	36 (34)	0.788	13 (38)	8 (38)	0.162	2 (67)	2 (67)	1.000
Cefuroxime	0 (0)	—	—	1 (33)	0 (0)	0.667	—	1(50)	—	—	—	—
Cefotaxime	0 (0)	0 (0)	—	34 (33)	32 (30)	0.628	10 (30)	5 (25)	0.495	1(50)	—	—
Ceftazidime	—	—	—	—	2(100)	—	—	—	—	—	—	—
Ceftriaxone	0 (0)	0 (0)	—	4 (44)	1 (50)	0.050	1 (100)	1 (100)	—	0(0)	—	—
Cefepime	—	—	—	—	—	—	—	—	—	—	—	—
Vancomycin	—	—	—	0 (0)	0 (0)	—	0 (0)	0 (0)	—	—	—	—
Gentamicin	7 (64)	1 (33)	0.376	32 (30)	20 (28)	0.835	4 (13)	1 (8)	0.664	2 (67)	2 (67)	1.000
Tobramycin	—	—	—	—	—	—	—	—	—	—	—	—
Amikacin	1(100)	—	—	0 (0)	0(0)	—	0 (0)	0 (0)	—	—	1 (100)	—
Netilmicin	—	—	—	0 (0)	—	—	—	—	—	—	—	—
Ciprofloxacin	1 (10)	3 (33)	0.216	34 (32)	27 (26)	0.377	11 (37)	6 (29)	0.538	2 (67)	2 (67)	1.000
Cotrimoxazole	5 (45)	4 (67)	0.078	32 (27)	16 (16)	0.063	3 (9)	1(5)	0.957	2 (67)	1 (100)	0.071

*n* = bacterial isolates among patients tested. Note: *p* values in bold indicate significant effects.

**Table 9:** Antimicrobial resistance pattern of Gram-negative isolates among two-year study period.

Antimicrobial agents	*Klebsiella* spp. (*n* = 63)			Acinetobacter spp. (*n* = 7)			*Pseudomonas aeruginosa* (*n* = 5)			*Pseudomonas* spp. (*n* = 12)			*Escherichia coli* (*n* = 42)			*Salmonella* spp (*n* = 4)		
	Resistance (%)			Resistance (%)			Resistance (%)			Resistance (%)			Resistance (%)			Resistance (%)		
	2016 (*n* = 25)	2017 (*n* = 38)	*p* value	2016 (*n* = 4)	2017 (*n* = 3)	*p* value	2016 (*n* = 3)	2017 (*n* = 2)	*p* value	2016 (*n* = 4)	2017 (*n* = 8)	*p* value	2016 (*n* = 17)	2017 (*n* = 25)	*p* value	2016 (*n* = 3)	2017 (*n* = 1)	*p* value
Ampicillin	24(96)	36(97)	**≤0.001**	4(100)	3(100)	—	—	—	—	—	—	—	13(76)	21(84)	**≤0.001**	1(33)	0(0)	0.667
Ampicillin/sulbactam	1(100)	15(100)	**≤0.001**	—	0(0)	—	—	—	—	—	—	—	—	1(100)	—	—	—	—
Augmentin	—	22(61)	—	—	1(100)	—	—	—	—	—	—	—	—	3(13)	—	—	0(0)	—
Piperacillin/tazobactam	3(100)	13(87)	**≤0.001**	3(100)	1(100)	—	0(0)	—	—	0(0)	—	—	—	1(100)	—	—	—	—
Cephalexin	19 (82)	30 (83)	**0.025**	4 (100)	1 (100)	—	—	—	—	—	—	—	9(69)	13(59)	**0.022**	—	(0)0	—
Cefuroxime	84	76	**0.004**	75	33	0.541	—	—	—	100	—	—	71	60	**0.018**	0	0	—
Cefotaxime	67	75	**0.032**	75	100	**0.010**	0	—	—	0	—	—	60	57	**0.005**	—	0	—
Ceftazidime	50	58	0.158	75	33	0.541	33	0	0.495	0	0	—	31	12	**0.041**	—	0	—
Ceftriaxone	68	70	**0.010**	75	100	**0.010**	0	—	—	0	—	—	65	57	**0.046**	0	0	—
Cefepime	100	94	**≤0.001**	100	100	—	0	—	—	0	—	—	—	100	—	—	—	—
Imipenem	33	38	0.067	33	100	0.056	0	—	—	0	—	—	—	100	—	—	—	—
Meropenem	25	93	0.604	100	100	—	—	—	—	—	—	—	—	100	—	—	—	—
Gentamicin	55	47	0.656	75	100	**0.010**	33	50	0.995	33	25	0.704	20	8	0.320	—	—	—
Tobramycin	—	—	—	—	—	—	33	50	0.995	0	25	0.389	—	—	—	—	—	—
Amikacin	20	39	0.338	75	33	0.541	33	50	0.995	33	0	0.104	0	8	0.242	0	0	—
Netilmicin	29	45	0.685	75	33	0.541	33	50	0.995	33	0	0.104	6	8	0.934	—	0	—
Ciprofloxacin	59	60	**0.034**	75	100	**0.010**	33	0	0.495	0	25	0.389	50	41	**0.038**	0	0	—
Cotrimoxazole	50	—	—	—	—	—	—	—	—	—	—	—	50	100	0.333	—	—	—
Colistin	0	7	0.668	0	0	—	—	—	—	—	—	—	—	—	—	—	—	—

*n* = bacterial isolates among patients tested. Note: *p* values in bold indicate significant effects.

**Table 10:** Multidrug resistance (MDR) isolates for the two-year study period.

Bacterial isolates	Number (%)	Year		*p* value
		2016 *n* = 239	2017 *n* = 232	
Coagulase-negative *Staphylococcus* (CoNS)	62 (45.2)	37 (52.1)	25 (37.8)	**≤0.001**
*Staphylococcus aureus*	10 (7.2)	8 (11.2)	2 (3.0)	**≤0.001**
*Enterococcus* spp.	4 (3.0)	2 (2.8)	2 (3.0)	0.182
*Klebsiella* spp.	43 (31.3)	15 (21.1)	28 (42.4)	**≤0.001**
*Acinetobacter* spp.	4 (3.0)	3(4.2)	1 (1.5)	0.194
*Pseudomonas aeruginosa*	1 (0.7)	1 (1.4)	0 (0.0)	—
*Escherichia coli*	13 (10.0)	5 (7.0)	8 (12.1)	**≤0.001**
Total MDR isolates	137	71	66	

Note: *p* values in bold indicate significant effects.

## Data Availability

The datasets used and/or analyzed during the current study are available from the corresponding author on reasonable request.

## Abbreviations

BSI: Bloodstream infections
DIC: Disseminated intravascular coagulation
CLSI: Clinical and Laboratory Standards Institute
CoNS: Coagulase-negative *Staphylococcus*
MDR: Multidrug resistance.
